# Artery tertiary lymphoid organs, neuro-immune interaction and their mediators in atherosclerosis

**DOI:** 10.1007/s00395-026-01164-x

**Published:** 2026-03-03

**Authors:** Silvia Ortona, Caterina Ivaldo, Luca Liberale, Federico Carbone, Fabrizio Montecucco, Martina Bastianon, Maddalena Mastrogiacomo, Domenico Palombo, Giovanni Pratesi, Chiara Barisione

**Affiliations:** 1 Department of Experimental Medicine, University of Genoa, Genoa, Italy; 2 Department of Surgical and Integrated Diagnostic Sciences, University of Genoa, Genoa, Italy; 3 IRCCS Azienda Ospedaliera Metropolitana, Genoa, Italy; 4 Department of Internal Medicine and Medical Specialities, University of Genoa, Genoa, Italy; 5 Clinic of Vascular and Endovascular Surgery, IRCCS Azienda Ospedaliera Metropolitana, Genoa, Italy; 6 Biotherapy Unit, IRCCS Azienda Ospedaliera Metropolitana, Genoa, Italy; 7 Research Center for Biologically Inspired Engineering in Vascular Medicine and Longevity, University of Genoa, Via Montallegro, 1, 16145 Genoa, Italy

**Keywords:** Atherosclerosis, Artery tertiary lymphoid organs (ATLOs), Neuro-immune interaction, Carotid stenosis, Abdominal aortic aneurysm, Peripheral nervous system

## Abstract

Atherosclerosis is a chronic inflammatory disease characterized by the irreversible remodeling of the arterial wall; severe atherosclerotic lesions may lead to life-threatening consequences such as major ischemic events (i.e., myocardial infarction (MI) and stroke) and abdominal aortic aneurysm (AAA) rupture. The severity of the lesions is determined by multiple risk factors that cause systemic and cellular metabolic changes, oxidative damage, cell senescence, and immune activation involving both leukocytes and vascular cells. In advanced stages, macrophage infiltration, alterations of the load-bearing collagenous matrix, and the presence of microcalcifications are the main drivers of plaque vulnerability. Over the last decade, the presence of artery tertiary lymphoid organs (ATLOs) has been established. These structures form during progressive atherosclerosis in the adventitia of large arteries and represent highly organized niches composed of T and B lymphocytes and innate immune cells. More recently, the presence of nerve fibers and the contribution of both the central (CNS) and peripheral (PeriphNS) nervous systems, through the action of sympathetic, parasympathetic, and somatosensory pathways regulating ATLO composition have been demonstrated. However, their role in atherosclerosis progression remains debated. This review explores the architecture of ATLOs and their neuroimmune interactions with the spleen, as a central neuroimmune organ, in atherosclerosis progression, with a particular focus on carotid stenosis and AAA. Furthermore, it highlights the neuronal mediators that could act as biomarkers of plaque instability and promising pharmacological targets. Finally, while still in the preclinical phase, it explores future prospects for integrating neuroimmune-based therapies into current clinical management of atherosclerosis.

## Introduction

Atherosclerosis is a chronic disease and a leading cause of major cardiovascular events such as MI and ischemic stroke. Its pathogenesis is sustained by multiple modifiable and non-modifiable risk factors and proceeds through complex interactions between lipid accumulation, chronic inflammation, structural cell activation, and eventually vascular dysfunction [56]. Impairment of the nitric oxide (NO) balance, increase of oxidative stress, and overexpression of adhesion molecules enable the recruitment of monocytes into the intima [23]. Once inside the vessel, these monocytes transform into macrophages and form foam cells, marking the onset of plaque development [97]. Foam cells release pro-inflammatory cytokines (e.g., tumor necrosis factor-alpha (TNFα), IL-1β, IL-6), amplifying inflammation and recruiting additional immune cells, further perpetuating the disease [57, 82]. Then, smooth muscle cells (SMCs) proliferate and migrate to the intima, contributing to the formation of the plaque [27, 56].

Traditionally, the “inside-out” paradigm has explained atherosclerosis as an inflammatory response originating in the intima due to endothelial injury and lipid oxidation. Later on, the “outside-in” paradigm has gained attention, looking at the adventitia and the perivascular visceral fat as potential sites where inflammation begins. In this model, adventitial fibroblasts undergo a phenotypic switch, producing inflammatory mediators and promoting vasa vasorum neovascularization. These processes propagate inflammation inward through the arterial wall [25, 64]. Both paradigms emphasize the central role of inflammation, which drives the progression of atherosclerotic plaques. When developing within the carotid artery, the atherosclerotic plaque is a critical determinant of stroke risk, with features of instability such as intraplaque hemorrhage, thin or ruptured fibrous caps, and extensive inflammation significantly increasing vulnerability to rupture and thromboembolism [86].

In advanced atherosclerosis, several mechanical, cellular, and molecular peculiarities associate with inflammatory profile and lesion complexity, leading to unstable plaque when hemodynamic forces exceed the resistance of the fibrous cap. Jansen and colleagues argued that three major players eventually determine the degree of plaque vulnerability: the load-bearing collagenous matrix, which confers protection, macrophages, responsible for extracellular matrix (ECM) degradation, and microcalcifications, as focal stress convectors and determinants of intraplaque vulnerable sites [47] addressing hypotheses for risk stratification improvement and therapeutic strategies.

Mechanics ECM descriptors such as strength, elasticity, stress, and stiffness may also be affected by metabolic conditions, such as the formation of Advanced Glycation End products (AGEs) in metabolic, atherosclerotic-prone diseases. In human carotid endarterectomies, it has been demonstrated that AGEs accumulate predominantly in macrophages surrounding the necrotic core and co-localize with markers of apoptosis such as cleaved caspase-3 [34].

Although calcifications are a frequent finding in atherosclerotic lesions, irrespective of their vulnerability, differences in terms of dimension and distribution have been observed between stable and unstable carotid plaques, with macrocalcifications conferring resistance and associated with a M2 macrophage polarization in the former, and microcalcifications more abundant and associated with inflammation in the latter [75]. Moreover, it has been observed a reduced calcification associated with the presence of CD163 macrophages in high-risk plaques [87].

In this scenario, vascular smooth muscle cells (VSMCs) senescence has been demonstrated to promote the release of pro-inflammatory mediators, ECM and collagen imbalance and to be associated with plaque VSMCs and telomeric repeat-binding factor-2, suggesting prevention of senescence as a potential target for intervention [101]. More recently it has been demonstrated that inhibition of dipeptidyl peptidase 4, a serine protease overexpressed in senescent VSMCs from atherosclerotic lesions, counteracts the release of pro-inflammatory and pro-thrombotic factors and increases senescent cell death, improving the overall vascular performance [39]. Spatial lipidomics of specimens retrieved during carotid endoarterectomy from both symptomatic and asymptomatic patients demonstrated an association between lipid content and plaque vulnerability, revealing that macrophages from symptomatic plaques are enriched in phosphatidylcholines, whereas the fibrous cap in asymptomatic plaques contains mainly lysophosphatidylcholines and cholesteryl esters, known to promote VSMC proliferation and migration [31]. Moreover, it has been demonstrated that a first ischemic attack, per se, can trigger the recurrence of events, through inflammasome activation by neutrophil extracellular trap-derived cell-free DNA, suggesting inflammasome inhibition as treatment in the post-stroke recovery [12].

As discussed by Gianopoulos and colleagues [26], differences in terms of infiltrating immune cells’ abundance and lineage have been demonstrated in carotid arteries retrieved from symptomatic patients, not only in respect to the timing of surgical intervention after the ischemic events but also to the severity of the symptoms. Moreover, when considering the heterogeneity in plaque-derived macrophages, the gene expression of inflammasome pathway and foam cell formation is higher in asymptomatic patients, while for iron metabolism and storage related genes, the profile committed towards clearance of iron and metabolites from intraplaque hemorrhage and healing after plaque rupture is higher in symptomatic patients [26].

Metabolic disorders, such as obesity, dyslipidemia, diabetes, and hypertension, not only promote atherosclerosis as risk factors predisposing to endothelial dysfunction and inflammation but also initiate hypoxia-dependent intracellular metabolic shifts from mitochondrial oxidative phosphorylation to cytoplasmic anaerobic glycolysis [2, 7]. In white blood cells, this event induces reprogramming of cytokine expression and immune potential, exacerbating the differentiation into a pro-inflammatory profile [99]; this priming is supposed to occur in the bone marrow, in myeloid progenitor cells, originating innate immune cells trained to be in an “alert” mode, with a prolonged life span and able to give birth to clonal hematopoiesis, which have been found to associate with cardiovascular conditions mainly in the elderlies [2].

The cardiovascular system is closely connected to the lymphatic system, which comprises lymphoid organs, lymph nodes, and unidirectional absorptive vessels. Together, they constitute the circulatory system, which is responsible for the maintenance of fluid homeostasis, lipid transport, inflammation, and immune regulation in both health and disease [5]. Accordingly, chronic inflammatory conditions such as atherosclerosis are characterized by impaired lymphatic function and abnormal morphology [51], as observed in experimental mouse models of atherosclerosis [66] and MI [50].

Moreover, the interplay between the two systems in atherosclerosis is also detectable at the systemic level. In a prospective study comprising a large cohort of middle-aged and elderly individuals, significant associations were identified between circulating IL-4-producing T cells and the so-called “intermediate monocytes” and the incidence of ischemic stroke [21].

Consistently, perturbation of the balance between myeloid and lymphoid inflammatory components following short-term administration of immune checkpoint inhibitors has been shown to aggravate plaque inflammation and increase the necrotic core of atherosclerotic lesions [80].

Thus, investigating the contribution of lymphatic components may offer novel perspectives to counteract the progression of atherosclerosis and potentially other chronic inflammatory diseases [51].

### Basics of lymphoid organs organization and ATLO definition

Primary lymphoid organs (PLOs), such as thymus and bone marrow, are the anatomical sites where lymphocytes develop and acquire immunocompetence; secondary lymphoid organs (SLOs), including lymph nodes, spleen and mucosa-associated lymphoid tissues, provide a pre-organized microenvironment for antigen presentation and adaptive immune activation [78]. Their architecture is defined by highly specialized stromal cells, particularly fibroblastic reticular cells (FRCs), which generate the ECM scaffold, conduit systems and chemokine gradients that establish T cell zones, B cell follicles, follicular dendritic cell (FDC) networks, and perivascular niches [17]. FRCs subsets also orchestrate the spatial cues required for leukocyte positioning through constitutive production of CXCL13, CCL19 and CCL21, ensuring efficient T-B cell collaboration and immune cell homeostasis [17, 78].

Within SLOs, B cell maturation follows a stereotyped sequence: naïve B cells localize to follicular and marginal zones, interact with antigen-presenting cells, and, upon activation, enter germinal centers where they undergo somatic hypermutation, affinity-based selection in the light zone, and differentiation into plasma cells or memory B cells [45]. These processes rely on the structural integrity of FDC networks, stromal fibroblast scaffolds, and chemokine-dependent organization of dark and light zones, emphasizing the stromal-immune crosstalk that defines SLO functionality [17].

Unlike PLOs and SLOs which, with the exception of specific mucosal tissue [8], are congenital and constitutive structures [78], tertiary lymphoid organs (TLOs) form de novo in non-lymphoid tissues under conditions of chronic inflammation [4, 85] including autoimmune disorders (e.g., rheumatoid arthritis, Sjögren’s syndrome) and persistent infections [118].

Although not encapsulated, TLOs recapitulate key structural and functional features of SLOs, such as the presence of high endothelial venules (HEVs), lymphangiogenesis, organized B-cell follicles and T cell zones, germinal centers (GC) formation, and FDC differentiation [78].

TLOs support the generation of adaptive immune responses by promoting the selection and maturation of high-affinity B cells and the activation of T cells. Chemokines such as CXCL13, CCL19, and CCL21 play a central role in TLO development by recruiting lymphocytes and organizing them into distinct functional compartments [78]. In chronic inflammatory diseases, TLOs may contribute either to the resolution or the persistence of inflammation, depending on the balance between pro-inflammatory and regulatory signals within these structures [111, 118]. For instance, TLOs have been shown to associate with the progression of kidney damage and to be regulated by IL-17, highlighting their potential as therapeutic targets in kidney injury [61].

Recently, different teams of research highlighted the role of TLOs [118] and their arterial counterparts (ATLOs) [111], and atherosclerotic plaque TLO (PTLO) [53], in coordinating immune responses within atherosclerotic plaques [28].

ATLOs form in the adventitia of large arteries during the advanced stages of atherosclerosis and resemble SLOs in their architecture.

They include distinct zones for T and B lymphocytes, as well as germinal centers where B cells undergo somatic hypermutation and produce high-affinity antibodies; their maturation proceeds through defined stages, from early leukocyte aggregates to fully organized lymphoid microstructures containing B cell follicles and germinal center-like clusters, T cell areas, FDC networks, postcapillary HEVs, characterized by cuboidal endothelial cells enabling selective recruitment of naïve and central memory lymphocytes from the bloodstream into organized lymphoid structures [78, 111], and expanded adventitial lymphatic vessels [111], represented by endothelial channels responsible for draining tissue fluid and regulating the exit of leukocytes from peripheral tissues to draining lymphoid organs [110].

The adventitial lymphatic network plays a crucial role in regulating antigen drainage, leukocyte trafficking, and inflammatory resolution, and undergoes extensive remodeling during plaque progression [5, 110]. Thus, the minimal criteria to define an ATLO include lymphoid compartmentalization, presence of HEVs, lymphangiogenesis, FDCs, and lymphoid-organogenic chemokine expression; disorganized adventitial infiltrates lacking these features should not be labeled as ATLOs [111].

**Fig. 1:**
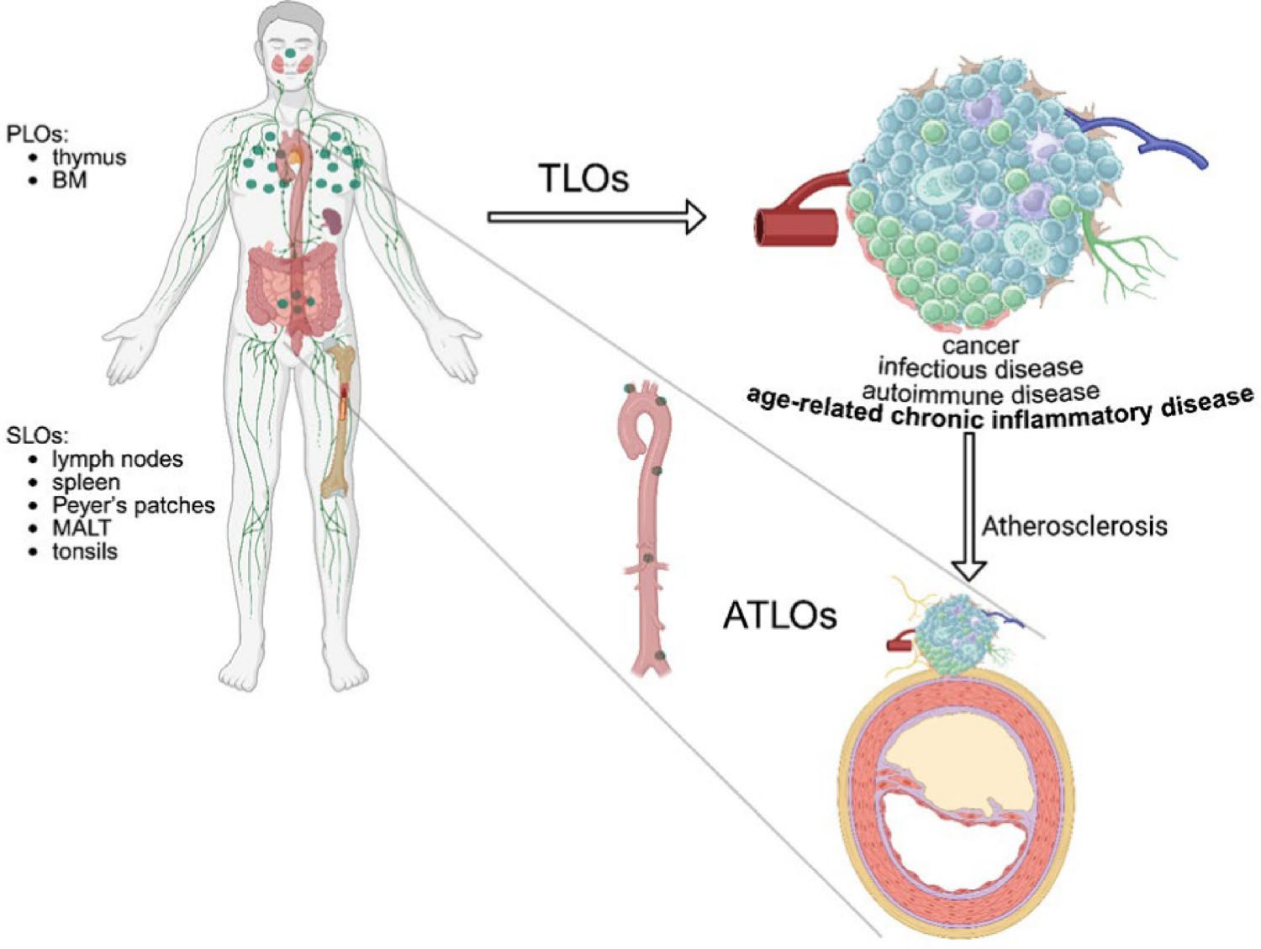
Anatomical localization of primary, secondary, and tertiary lymphoid organs and ATLOS (Created in BioRender. Montecucco, F. (2026) https://BioRender.com/x3a0osm)

Finally, ATLOs form within the neurovascular niche of the arterial adventitia, a region enriched in sympathetic and sensory nerve fibers. This preserved innervation is not a general feature of TLOs in other organs and may uniquely facilitate neuroimmune interactions contributing to ATLO development and function in atherosclerosis [111] (BOX 1; Fig. 1).

This review article explores the emerging role of ATLOs and neuroimmune interactions in the pathogenesis of atherosclerosis, with a particular focus on carotid stenosis and AAA progression. By examining the mechanisms driving ATLO formation and their contribution to plaque vulnerability, we highlight how targeting immune responses, potentially through neuronal pathways such as vagus nerve stimulation (VNS) or β-adrenergic blockade, may complement traditional lipid-lowering and anti-inflammatory therapies to reduce cardiovascular risk and prevent major complications such as ischemic events and AAA rupture.

BOX 1. Key concepts on lymphoid organs and the identity of ATLOs PLOs: thymus and bone marrow

Structure: organized into stromal niches formed by epithelial and mesenchymal cells, which provide necessary survival, selection, and differentiation signals for T- and B-cell maturation.

Function: sites of lymphocyte development and central tolerance.

SLOs: lymph nodes, spleen, tonsils, and mucosa-associated lymphoid tissues

Structure: constitutively formed during embryogenesis and maintained through stromal–immune crosstalk; a scaffold is created by FRCs; chemokine gradients and conduit networks for leukocyte trafficking. Pre-organized architecture with B and T cell zones, FDCs, HEVs, and lymphatic vessels.

Function: B cell maturation and differentiation into memory B cells or antibody-producing plasma cells.

**TLOs: ectopic aggregates**

Structure: ectopic lymphoid aggregates that form de novo in chronically inflamed, non-lymphoid tissues (i.e., autoimmune diseases, infectious diseases, organ transplantation, neoplasia). Nonencapsulated local hubs of adaptive immunity, they recapitulate SLOs for lymphoid chemokine expression (CXCL13, CCL21), HEV formation, lymphangiogenesis, B-/T-cell compartmentalization, and FDC development.

Function: elicit both protective or pathologic immune responses on the basis of the tissue microenvironment and their composition.

**ATLOs: adventitia of atherosclerotic arteries**

Structure: develop from early aggregates into lymph node-like structures featuring B cell follicles, T cell zones, FDC networks, HEVs, and expanded adventitial lymphatic networks.

Function: Unlike most TLOs in peripheral tissues, ATLOs form within the neurovascular niche of the arterial adventitia supporting neuroimmune interactions.

## Formation and role of ATLOs in atherosclerosis

### Development of ATLOs in atherosclerosis

ATLOs are a specialized form of TLOs that develop in the adventitia of large arteries, particularly in the context of chronic vascular inflammation seen in advanced atherosclerosis [43]. ATLOs develop in response to persistent antigenic stimulation, typically from modified self-antigens such as oxidized Low-Density Lipoproteins (oxLDL) and apoptotic cells accumulating in the atherosclerotic plaque [118]. Within the adventitia, fibroblasts—considered as FRC-like cells [111], VSMCs, and endothelial cells—produce the chemokines necessary for lymphoid neogenesis, initiating the formation of ATLOs. They serve as local sites where antigen presentation and immune responses are orchestrated, contributing to the ongoing inflammation within atherosclerotic plaques [44, 107].

As ATLOs expand during the progression of atherosclerosis, they contribute to both plaque destabilization and immune regulation. The chronic antigenic stimulation within ATLOs results in the accumulation of immune cells that perpetuate the inflammatory process. However, the balance of pro-inflammatory and regulatory cells within ATLOs ultimately determines whether these structures promote plaque progression or, vice versa, activate regulatory immune responses that stabilize the fibrous cap [73].

The formation of ATLOs represents a point of convergence between innate and adaptive immunity: innate immunity cells, such as macrophages and DCs, not only initiate the response, but also provide essential signals for the formation of GC and the maturation of T and B lymphocytes. Indeed, ATLOs are made up of B cell follicles, areas rich in T lymphocytes and dendritic cells (DCs) derived from monocytes, thus favoring lymphocyte activation and differentiation; experimentally, in atherosclerotic apolipoprotein E-deficient (ApoE-/-) mice, aging is associated with the progressive development of ATLOs into hubs for B-cell differentiation characterized by an autoreactive cellular profile [92].

Within the adaptive component subpopulations with opposite functions are generated: effector cells (CD4⁺, CD8⁺) that amplify inflammation, and regulatory cells (nTreg, iTreg, B-1) that promote tolerance. This dichotomy reflects the dual role of ATLOs, where innate and adaptive responses are intertwined, the balance of which is decisive in maintaining silent autoimmunity or, on the contrary, in promoting the clinical progression of atherosclerotic disease [107].

Of interest, immune cell recruitment and cytokine production within ATLOs are tightly controlled by the CNS and PeriphNS, particularly through the autonomic nervous system (ANS) [102]. The ANS plays a dual role in plaque severity progression: the sympathetic nervous system (SNS) promotes inflammation via norepinephrine (NE) release, which activates β-adrenergic receptors (ARs) and increases pro-inflammatory cytokines and matrix metalloproteinases (MMPs) activity, contributing to plaque destabilization. In contrast, the parasympathetic nervous system (PNS), mainly through vagus nerve signaling, reduces inflammation via the cholinergic anti-inflammatory pathway. Notably, the artery-brain circuit (ABC) [71] integrates neural and immune signals to modulate immune responses within ATLOs.

### Immune architecture of ATLOs

The interplay between the innate and adaptive immune systems in the context of atherosclerosis and chronic inflammatory diseases becomes particularly significant when examining the role of ATLOs [73, 111].

ATLOs represent highly organized immune niches, including areas of T cells, B cell follicles with active GC, and plasma cell niches, supported by neoangiogenesis, lymphangiogenesis, and high formation of endothelial venules. Through this complex architecture, comprehending specialized microanatomical structures such as lymphatic conduits, which allow antigen transport and immune cell interaction across arterial layers [73, 111], ATLOs orchestrate both protective and pathogenic immune responses, depending on the balance between effector and regulatory lymphocyte subsets [107].

The innate immune system serves as the first responder, recognizing and reacting to danger signals such as oxLDL or arterial wall stress. Cells like macrophages, DCs, and SMCs in the arterial wall are key players, producing cytokines and chemokines like CXCL13 and CCL21, which recruit T and B cells to the adventitia [48, 73, 111].

In aged ApoE-/- mice, SMCs in the medial layer beneath plaques activate lymphotoxin-β receptor (LTβR) signaling to initiate ATLO formation [30]. These structures are vital for localizing and amplifying immune responses in the arterial wall, showing features such as GCs, follicular DCs, and HEVs, which facilitate immune cell recruitment and retention [30, 48].

The adaptive immune system is subsequently engaged through antigen presentation by innate cells like DCs. These cells process antigens derived from arterial plaques and present them to naïve T cells, promoting the differentiation of T cell subsets within ATLOs. In the context of atherosclerosis, T follicular helper (Tfh) cells in GCs regulate B cell maturation and antibody production, including antibodies targeting oxLDL. These antibodies can contribute to both protective and pathogenic immune responses, depending on their specificity. In some cases, they may help clear modified lipids and apoptotic cells, while in others, they may exacerbate inflammation by forming immune complexes that activate complement [16, 111].

Importantly, the activity of the Tfh-GC B cell axis is tightly controlled by Qa-1-restricted CD8+ regulatory T cells, which act as critical brakes on excessive GC responses. Disruption of this regulatory pathway in ApoE-/- mice results in hyperactivation of Tfh-B cell interactions, accelerated ATLO formation, and worsened plaque development. Conversely, restoring this balance, for example, through Inducible T-cell Costimulator and its ligand (ICOS-ICOSL) blockade, limits TLO expansion and mitigates atherosclerosis progression [16].

In addition, regulatory T cells (Tregs) and follicular regulatory T (Tfr) cells within ATLOs contribute to immune modulation. Their balance against pro-inflammatory subsets such as Tfh1 and Tfh17 is crucial for maintaining immune homeostasis. Recent comparative studies underscore how shifts in these subsets influence vascular pathology. For instance, Kasashima and collaborators demonstrated that in IgG4-related abdominal aortic aneurysm (IgG4-AAA), ATLOs are unusually large and irregular, enriched in Tfr and Tfh2 cells, whereas atherosclerotic AAAs are characterized predominantly by Tfh1 expansion, and Takayasu arteritis by Tfh17 accumulation. Thus, an imbalance of Tfr/Tfh ratios emerges as a disease-specific immunological signature that shapes the morphology and function of ATLOs [48].

Gräbner and colleagues showed that medial SMCs, via LTβR signaling, upregulate CXCL13 and CCL21, thereby initiating ATLO formation in aged ApoE-/- mice. This process generates a fully organized lymphoid architecture, including B cell follicles with GC, HEVs, plasma cell niches, and functional conduit mesh works that connect arterial layers and enable local antigen transport [30]. Such a dynamic organization enhances the coordination between innate-driven inflammation and adaptive immune specificity, providing both protective and pathogenic potential. Dysregulation in this interplay, whether through excessive Tfh-driven antibody responses, altered Tfr/Tfh balance, or defective LTβR-dependent signaling, can amplify chronic inflammation, promote adventitial vasculitis, and ultimately contribute to plaque progression and instability [30, 111].

Very recently, Lai and colleagues presented their results about a spatially resolved single-cell transcriptome atlas of human atherosclerotic plaques; they provided evidence of TLO-like formations within carotid PTLOs, characterized by a poor stage of organization, absence of GCs, and presence of fibroblast-like SMCs, with the potential to recruit B cell aggregates. The B cell secreted IgG antibodies may elicit the macrophage effector functions; both PTLOs and IgG-positive plasma cells within the plaques are more abundant in specimens retrieved from symptomatic rather than asymptomatic patients, suggesting their contribution to disease severity [53].

In conclusion, the innate immune system initiates responses and facilitates the recruitment and activation of adaptive immune cells within ATLOs. These TLO are critical sites where antigen-specific responses are refined and sustained. The balance between pro-inflammatory and regulatory pathways within these structures dictates whether the immune response resolves inflammation or drives disease progression, underscoring the complex synergy between innate and adaptive immunity in vascular pathologies [30, 48, 73, 111].

### ATLOs and plaque vulnerability

The contribution of ATLOs in promoting plaque vulnerability is still debated, as these structures can either lead to plaque progression or, on the contrary, to plaque stabilization.

Indeed, the final result depends on the heterogeneity of both innate and acquired immune cells, their interaction with resident cells, and the balance among inflammatory and neuronal mediators.

When considering the macrophage population in the early stages of atherosclerosis, it is mainly represented by circulating monocyte-derived cells, while in mature lesions, resident, proliferating macrophages are present. In ApoE-/- mice, the investigation of atherosclerotic lesions revealed four subsets of macrophages, different in transcriptome, phenotype, shapes, sizes, movements, and migration in the atherosclerotic artery wall in vivo*.* Moreover, the comparison between different stages of atherosclerosis revealed differences between the macrophage population in aortas with ATLOS, both in terms of transcriptome, phenotype, a dendritic cell-like shape, and function, compared with those without ATLOS [67].

The activated immune cells secrete cytokines like IFN-γ, TNF-α, and IL-17, which drive the recruitment of additional immune cells, increase vascular inflammation, and stimulate the production of MMPs [23, 111]. MMPs are enzymes that degrade the ECM, including the collagen fibers that form the fibrous cap of the atherosclerotic plaque. The thinning of the fibrous cap due to MMP activity increases the risk of plaque rupture, which can lead to thrombus formation and subsequent ischemic events such as MI and stroke [23]. Of note, IL-7r is differentially expressed in the aged ApoE-/- aorta and ATLOs of atherosclerotic mice, and is thought to orchestrate interaction between cytokine, their receptors, and downstream pathways toward a pro-inflammatory profile. This observation suggests IL-7r as a driver toward disease progression and ATLO lymphogenesis and development [116].

Conversely, Tregs within ATLOs can promote plaque stability by suppressing excessive immune activation and limiting the production of pro-inflammatory cytokines and MMPs. Tregs secrete IL-10 and TGF-β, which inhibit the activity of pro-inflammatory immune cells and promote the production of ECM proteins that reinforce the fibrous cap. The presence of Tregs within ATLOs has been associated with reduced inflammation and increased plaque stability, suggesting that enhancing Treg function could be a therapeutic strategy to prevent plaque rupture [111].

### ATLOs and abdominal aortic aneurysms

AAAs consist of a permanent dilation of the aorta due to degeneration of the arterial wall and are considered a complication of progressive atherosclerotic lesions; ATLOs have been observed in advanced AAAs and are thought to play a role in disease severity. Studies have identified the presence of ATLOs in the adventitia of aneurysmal aortas, suggesting their involvement in local immune responses that may influence aneurysm development and expansion [20]. The cellular composition of ATLOs in aneurysms includes various immune cells such as T and B lymphocytes, DCs, macrophages, and mast cells. Notably, immunoglobulin-producing B cells within these structures have been linked to aneurysmal progression. For instance, an amplification loop involving IgE, mast cells, and B cells has been implicated in AAA progression, where local IgE production by B cells can activate mast cells, leading to the release of inflammatory mediators that exacerbate vascular damage [60]. Furthermore, ATLOs have been associated with specific microRNA expression profiles in AAA tissues. This study highlights the potential of ATLO-derived microRNAs, such as miR-15a-3p and miR-30a-5p, as biomarkers for AAA, offering novel insights into the pathophysiology and diagnostic strategies for this disease [95].

Another study highlights the critical role of ATLOs in IgG4-AAA, a disease marked by chronic inflammation and fibrosis of the abdominal aorta. ATLOs in IgG4-AAA are morphologically distinct, being larger, more numerous, and irregular compared to those observed in conditions such as atherosclerosis or Takayasu arteritis. These abnormalities correlate with elevated serum IgG4 levels and the presence of IgG4-positive plasma cells, linking ATLO features to disease activity. The study also identifies abnormal ATLOs enriched with Tfr cells as a potential pathological marker for IgG4-AAA diagnosis, particularly in cases where traditional markers such as serum IgG4 levels are inconclusive. In addition, targeting Tfr cells could represent a novel therapeutic avenue for managing this condition [48].

While the exact role of ATLOs in aneurysm pathophysiology remains to be fully elucidated, their presence indicates a localized immune response that could either contribute to disease progression or represent an attempt to control vascular inflammation. However, since ATLOs are widely represented in specimens retrieved from human and preclinical models of AAAs, they represent a useful platform of investigation providing insights to translate to other arterial districts [70].

Further research is needed to determine whether ATLOs have a protective or detrimental effect on aneurysmal diseases and to explore potential therapeutic strategies targeting these structures.

## Neuroimmune interactions in atherosclerosis

**Fig. 2:**
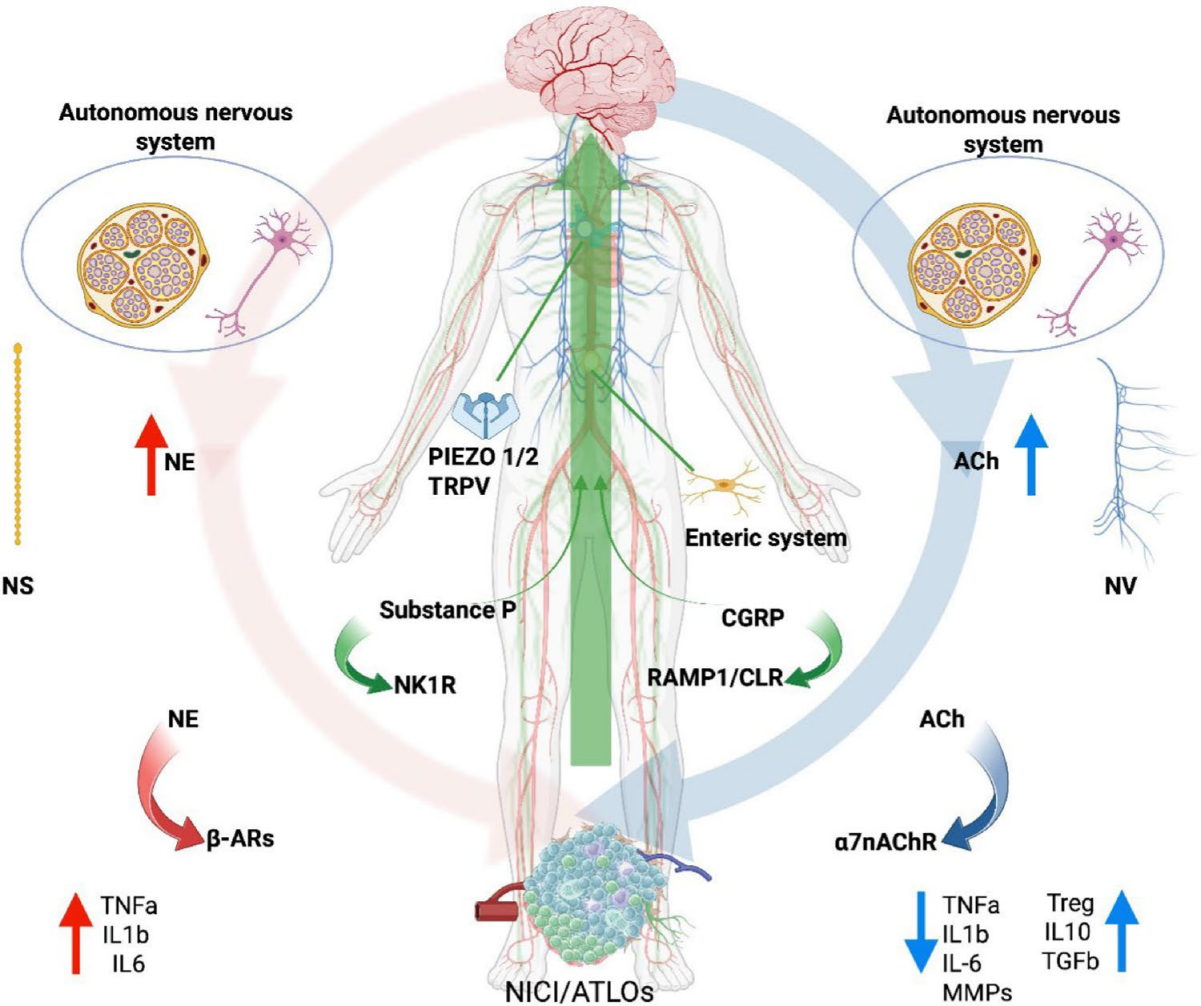
Interaction of Peripheral Nervous System components affecting the cardiovascular system Red—Sympathetic ANS: NE, β-ARs. Blue—Parasympathetic ANS: Vagus nerve (“*Nervus Vagus*”, NV), ACh, α7nAChR. Green—Sensory NS (somatosensory and enteric NS): mechanosensitivity ion channels (PIEZO 1,2), Transient Receptor Potential (TRP) channels; Substance P (SP); Neurokinin 1 Receptor (NK1R); Calcitonin Gene-Related Peptide (CGRP); Receptor Activity-Modifying Protein 1 and Calcitonin Receptor-Like Receptor (RAMP1/CLR). (Created in BioRender. Montecucco, F. (2026) https://BioRender.com/x3a0osm)

The cardiovascular–brain–immune (CBI) axis has changed our understanding of atherosclerosis, framing it not merely as a vascular disease but as a condition driven by complex neuroimmune interactions. Connections between the CNS, cardiovascular system, and immune responses maintain homeostasis under physiological conditions and regulate vascular inflammation and plaque progression in atherosclerosis [91] (Fig. 2). Within this framework, neuroimmune cardiovascular interfaces (NICIs) represent critical sites where the brain communicates with the immune system to maintain vascular homeostasis or, conversely, to exacerbate disease [70, 71, 74].

The ABC is a polysynaptic network connecting afferent and efferent neurons from the arterial wall to the CNS. In atherosclerosis, the arterial adventitia serves as a focal point of this axis, where ATLOs act as neuroimmune hubs. Sensory afferent neurons convey inflammatory signals from the plaque to the brain, whereas efferent sympathetic fibers sustain inflammation by activating pro-inflammatory immune cells through NE and β2-AR signaling [70]. In contrast, the PNS, primarily via the vagus nerve, mediates anti-inflammatory pathways through Acetylcholine (Ach)/ Alpha 7 nicotinic acetylcholine receptors (α7nAChR) activation on immune cells, promoting Th2/Treg responses and IL-10 secretion [91].

This circuit perpetuates inflammation by reinforcing sympathetic activity while modulating parasympathetic responses, which in turn influence local immune reactions within the arterial wall [72].

Importantly, innervation is not uniformly distributed throughout the arterial tree; different vascular segments exhibit distinct patterns of sensory [69] and autonomic [42] input, which contribute to shaping local neuroimmune reflexes and regulating the balance between pro- and anti-inflammatory mediators in both physiological and pathological conditions [70].

Understanding these mechanisms is particularly important in carotid stenoses, where unstable plaques correlate with the systemic immune-inflammation index, as calculated from peripheral blood-derived parameters, and are associated with an increased risk of thromboembolic events leading to ischemic stroke [115]. Furthermore, tertiary lymphoid-like structures identified in carotid endarterectomy specimens correlate with cerebrovascular events; these structures are proposed to act as local sites of IgG production, enhancing macrophage pro-inflammatory activity and contributing to plaque vulnerability [53].

### Sympathetic nervous system and pro-inflammatory signaling

The activation of the SNS has emerged as a central driver of inflammation in atherosclerosis [91]. Among various determinants, mental stress is a recognized cardiovascular risk factor, due to its ability to activate the SNS [49]. Using an acute stress mouse model, Hinterdobler and colleagues demonstrated that the release of NE, the most important neurotransmitter released by the postganglionic axon [42], promotes the recruitment of inflammatory leukocytes into atherosclerotic plaques and the endothelial activation to a much greater extent than epinephrine or corticosterone derived from the neurohormonal axis [42]. Moreover, analyses of human carotid endarterectomy specimens revealed increased levels of NE [42] and neuropeptide Y (NPY) [52], a co-transmitter in vascular sympathetic nerves [1], supporting a key role for sympathetic neurotransmission in human atherosclerotic disease.

Sympathetic nerves innervate the adventitia and release NE in response to inflammatory stimuli, binding to AR on immune cells, VSMCs, and endothelial cells. This neurotransmitter’s interaction with β-ARs on immune cells activates pro-inflammatory pathways, stimulating the production of cytokines such as TNF-α, IL-1β, and IL-6 [71, 72].

NE-driven signaling facilitates immune cell recruitment, driving macrophages and T cells into the atherosclerotic plaque. Once present, these immune cells secrete inflammatory mediators and further recruit additional immune cells, creating a vicious cycle that intensifies local inflammation. Notably, the upregulation of MMPs in response to sympathetic signaling contributes to plaque instability. MMPs degrade the ECM, weakening the plaque structure and increasing the risk of rupture, a particularly dangerous event in carotid arteries, where rupture can lead directly to thromboembolic events and stroke [71].

NE also regulates vascular tone primarily through α1-AR-mediated, Ca^2^⁺ dependent contraction of VSMCs [102]. Engagement of α1-ARs activates phospholipase C, generating inositol trisphosphate and triggering Ca^2^⁺ release from intracellular stores, as well as opening voltage-dependent L-type Ca^2^⁺ channels. The rising cytosolic Ca^2^⁺ binds calmodulin, which then activates myosin light chain kinase to induce VSMC contraction [59]. In mesenteric arteries, NE-induced contractility is finely tuned by intracellular Ca^2^⁺ dynamics, including Ca^2^⁺ waves and “sparklets,” which locally activate calcineurin, a Ca^2^⁺/calmodulin-dependent phosphatase critical for vascular reactivity. Pharmacological inhibition of calcineurin (e.g., cyclosporin A or FK506) impairs NE-induced contraction, disrupts cellular pH balance, and contributes to endothelial and VSMC dysfunction [77].

Calcineurin also promotes translocation of nuclear factor of activated T cells (NFAT) into the nucleus, inducing transcription of proliferative genes such as cyclin A and c-myc that culminate in VSMC hypertrophy and arterial remodeling. Dysregulation of the calcineurin-NFAT pathway leads to maladaptive hypertrophy, increased vessel stiffness, and elevated vascular resistance, contributing to pathological conditions such as hypertension and atherosclerosis [79].

### Parasympathetic nervous system and the cholinergic anti-inflammatory pathway

The ANS contributes to the formation of the ABC through a second efferent component, the parasympathetic nervous system (PNS). To date, a direct impact of PN fibers on arteries has not been demonstrated; instead, the PNS acts indirectly by innervating target organs, including the heart and visceral smooth muscles, and by modulating the immune system [112].

Murine models have not demonstrated the presence of parasympathetic axons in ATLOs; they have revealed the presence of ACh-positive leukocytes [70]. ACh is the principal neurotransmitter of the PNS and mediates effects that counteract inflammation and other responses promoted by the SNS.

The PNS regulates vascular inflammation primarily via the cholinergic anti-inflammatory pathway. When activated, the vagus nerve releases ACh, which binds to α7nAChR on macrophages and other immune cells, inhibiting the production of pro-inflammatory cytokines and promoting immune tolerance within atherosclerotic plaques [102].

ACh also regulates vascular tone and hemodynamic forces by activation of endothelial nitric oxide synthase (eNOS) and release of NO, which diffuses into VSMCs and stimulates guanylate cyclase, increasing cyclic GMP levels and promoting vasorelaxation. In addition, ACh influences the activity of large-conductance calcium-activated potassium channels in VSMCs, causing membrane hyperpolarization and further facilitating vessel relaxation [32]. The activation of these channels contributes to the overall reduction of arterial resistance and blood pressure, playing a protective role in cardiovascular homeostasis. Under inflammatory conditions, such as exposure to TNFα, ACh-induced vasodilation can be impaired due to oxidative stress, reduced NO bioavailability, and dysregulation of potassium channels, ultimately compromising vascular function and increasing vascular tone [15].

However, as discussed by Vieira-Alves and colleagues [100], the real contribution of α7nAChR activation is the result of complex pathways involving both neuronal and non-neuronal cholinergic systems. The latter mainly operate through autocrine/paracrine loops among immune cells, which respond differently by releasing either pro- or anti-inflammatory mediators depending on the specific cell population involved.

**Fig. 3:**
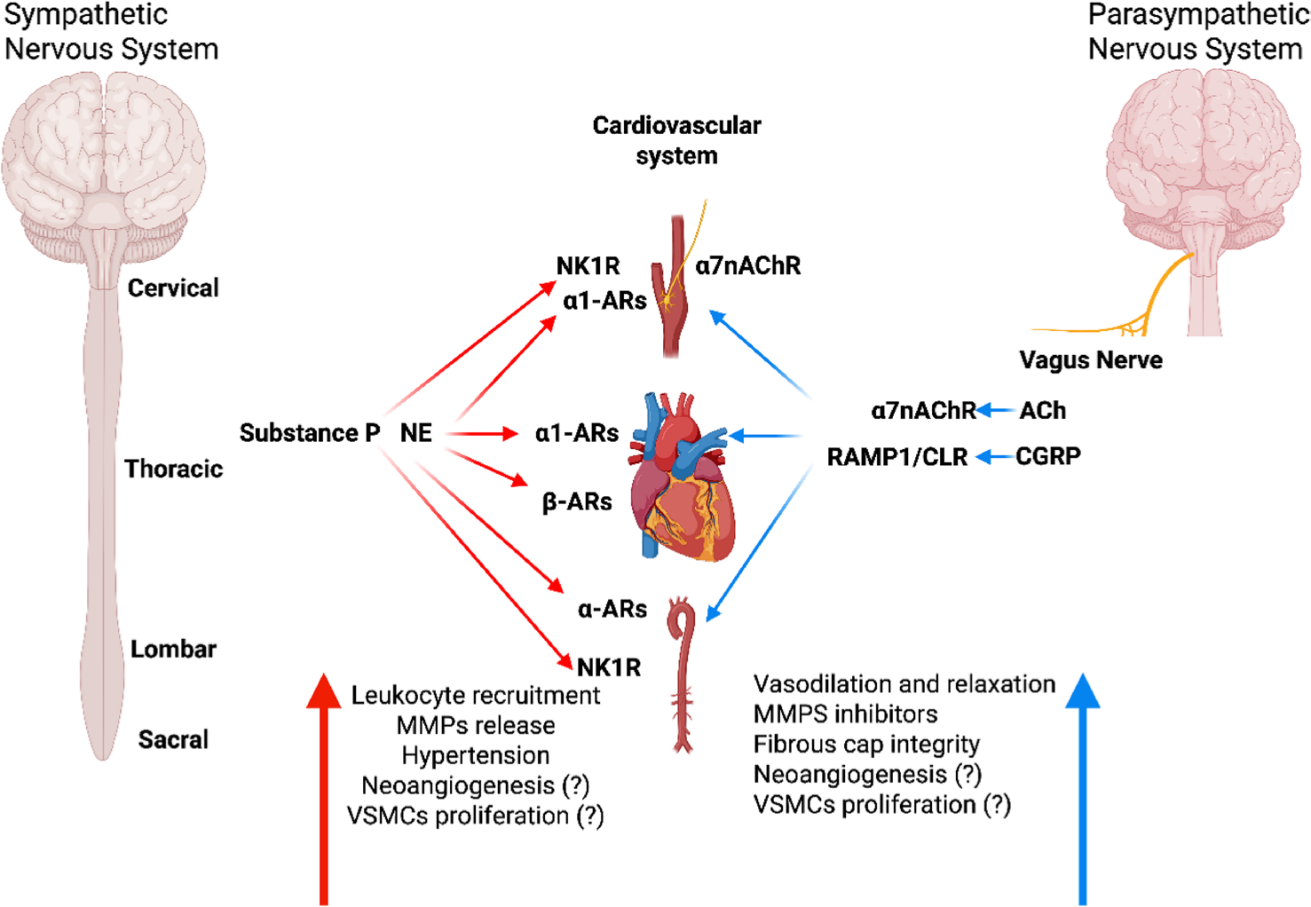
Effects of the ANS on the cardiovascular apparatus Sympathetic ANS (left side, red arrows): cell ganglia release NE, which directly activates α1-ARs, β-ARs on the arterial wall, heart, leukocytes, leading to their recruitment into the arterial wall. Parasympathetic ANS (right side, blue arrows): VN activation sustains the cholinergic pathway mainly by release of Ach, which activates α7nAChR present on the heart, arterial walls and leukocytes, leading to vasorelaxation and modulation of the inflammatory response. (Created in BioRender. Montecucco, F. (2026) https://BioRender.com/x3a0osm)

Moreover, the shift toward alternative immune polarization, although contributing to the attenuation of the inflammatory burst, may eventually promote the pro-angiogenic reprogramming of VSMCs and ECs. Indeed, as demonstrated in the context of carotid atherosclerosis, this process may result in intraplaque neovasa formation and plaque destabilization [33]. This multifaceted interplay, together with the intrinsic complexity of the lesion, should be taken in account when considering manipulation of the cholinergic system as a strategy to prevent major atherosclerotic events [100] (Fig. 3).

### Sensory nervous system and the afferent pathway of NICI/ATLOs

Within the adventitial–neuronal complex, the afferent branch connecting NICI with the CNS consists of a dense network of sensory fibers [54], mainly comprising primary nociceptive sensory fibers, unmyelinated C fibers, and thinly myelinated Aδ fibers. These fibers transduce inflammatory, noxious, and mechanical signals from the plaque microenvironment to the CNS [24]. During atherosclerotic progression, these fibers may release mediators that locally modulate immune responses and inflammation. Sensory fibers detect tissue stress, damage, or inflammatory mediators and transmit this information both to the CNS and to local effector circuits [76].

Primary sensory fibers are enriched in TRP channels, particularly TRPV1 (TRP vanilloid subfamily member 1), a cation channel with dynamic selectivity for H⁺, Na⁺, Ca^2^⁺, and Mg^2^⁺. TRPV1 can be activated by nociceptive thermal stimuli (temperature > 43 °C), weakly acidic environments (pH < 6.0), capsaicin, peptide toxins, arachidonic acid metabolites, and oxygenase products [117].

TRP channels, especially TRPV1, are also expressed in VSMCs and ECs, where they modulate vascular tone and exert protective effects against atherosclerosis by regulating oxidative stress, apoptosis, and inflammatory cytokine release. Pharmacological activation of TRPV1, for example via capsaicin, has been suggested as a potential therapeutic approach. In sensory neurons, TRPV1-mediated cation influx induces cell depolarization and action potential generation, which is transmitted to the CNS to encode nociceptive signals [114].

Thus, sensory nerves not only convey information to the CNS but also modulate vascular tone and release neuropeptides such as SP and CGRP, which influence immune cell recruitment, activation, and cytokine production, thereby promoting a local pro-inflammatory environment [1]. CGRP additionally restrains VSMC proliferation, reducing neointimal hyperplasia. Prolonged TRPV1 activation may induce receptor desensitization via calcium-dependent dephosphorylation, potentially diminishing its neuroprotective effects during chronic inflammation [24].

One of the first pieces of experimental evidence of sensory fiber involvement in pressure-mediated responses dates back approximately 20 years: vascular sensory C-fibers contribute to acute vasoconstriction upon increased intraluminal pressure, and this mechanosensitivity is mediated at least in part by TRPV1 [89].

In the context of atherosclerosis, TRPV1⁺ sensory fibers co-expressing CGRP within the NICI detect inflammatory cues and transmit them via dorsal root and nodose ganglia to autonomic centers in the brainstem, parabrachial nucleus, and hypothalamus [70]. Efferent sympathetic pathways then descend from these centers to the vascular adventitia via spinal and celiac ganglia, releasing NE to modulate leukocyte behavior and endothelial activation. Ablation of the celiac ganglion in aged atherosclerotic mice disrupts NICI integrity, attenuates local immune activation, and reduces plaque progression, highlighting the importance of this peripheral neural circuit in late-stage disease [70, 91].

Thus, within NICIs, the TRPV1–CGRP axis functions as a key sensory immunomodulatory pathway, where TRPV1 activation in perivascular sensory afferents triggers CGRP release in response to inflammatory sensitization.

More recently, another mechanosensitivity ion channels family, PIEZOs, comprising PIEZO1 and PIEZO2, has been recognized for its ability to keep vascular homeostasis and mediate vascular inflammatory responses [113].

PIEZO1 promotes EC inflammation in response to oscillatory shear stress [55] and enhances macrophage clearance within atherosclerotic plaques, reducing plaque size and necrotic core, increasing collagen content, and lowering the inflammatory profile [81].

PIEZO2 has been found abundantly expressed in a subset of sensory neurons innervating the aortic arch, where it detects stretch, blood pressure, and shear stress, contributing to real-time stabilization of cardiovascular output. Activation of PIEZO2⁺ neurons generates action potentials that convey mechanical information to the CNS and initiate local signaling within the aortic microenvironment [69]. By sensing abnormal mechanical forces, PIEZO2⁺ neurons can create a feedback loop that integrates mechanical and inflammatory cues, potentially affecting atherosclerosis, hypertension, and vascular homeostasis [69].

## Neuroimmune interactions and therapeutic implications in atherosclerosis

The conceptualization of atherosclerosis has shifted from a strictly vascular disorder to a complex neuroimmune pathology, where bidirectional communication between neurons and immune cells shapes plaque development and stability.

Growing evidence recognizes the spleen as a central organ for the neuroimmune control of cardiovascular function, with its vagal and sympathetic innervation involved in hypertension [13, 40] and other cardiovascular diseases. Indeed, the spleen is not only a central immune organ that has a relay function in circulating blood, but it is also connected to the autonomic nervous system and the somatosensory nervous system. In physiological conditions, the spleen serves as a site for maturation and storage of the bone marrow-derived myeloid cells that can be rapidly released if activated by damage-associated molecular patterns, as occurs in the case of MI or stroke. Therefore, the spleen is involved in the (sub)acute inflammatory infiltration of the infarcted myocardium via the release of peripheral immune cells [2].

As proven in an ischemia–reperfusion mouse model, the anti-inflammatory pathway initiated in the central nervous system is mediated by the spleen as follows: efferent vagal activation of the noradrenergic splenic nerve, in the celiac and suprarenal ganglia induces β2-AR activation of T cells in the spleen, which contain choline acetyltransferase and release of Ach. In turn, Ach activates α7nAChR in splenic macrophages to reduce the release of pro-inflammatory cytokines [46].

Preclinical models suggest immune modulation through stimulation of the autonomic connections to the spleen as a novel therapeutic strategy for the treatment of inflammatory conditions. In pigs, splenic nerve stimulation was found to promote cardiovascular protection as well as cytokine modulation using high and low doses of lipopolysaccharide as a pro-inflammatory challenge [18].

By contrast, excessive sympathetic activation, as occurs during the stroke, induces leukocyte release from the spleen that infiltrate the infarcted brain and exacerbate signs of myocardial injury. Rodent models demonstrate that the inflammatory response to such events is associated with inflammasome activation by circulating cell-free DNA, sustaining a feedforward loop towards atherosclerotic plaque destabilization. Splenectomy before or right after cerebral artery reperfusion reduces the cerebral infarct size, while its delay 3 days after stroke is no longer protective [14].

Recent studies have identified distinct neuronal markers, such as growth-associated protein 43 (GAP-43) and SP, and immune mediators, including CXCL13, IL-17, and α7nAChR, within human and experimental lesions, underscoring their roles in modulating local inflammation and cellular recruitment [106]. These molecules not only deepen our understanding of disease mechanisms but also serve as promising biomarkers for plaque vulnerability and as targets for precision interventions.

Ectopic tertiary lymphoid structures in the adventitia emerge as focal points where sensory and sympathetic fibers intersect with organized immune cell aggregates, creating a microenvironment that perpetuates cytokine release and antigen presentation. By profiling the neuroimmune signature of ATLOs, such as nerve density, neurotransmitter receptor expression, and chemokine gradients, researchers can develop diagnostic imaging probes (e.g., positron emission tomography (PET) tracers for neuroinflammation) and quantify treatment responses to neuromodulatory therapies. Therapeutically, this integrated perspective paves the way for novel strategies: targeted blockade of neuropeptide signaling to dampen adventitial inflammation; selective agonism of the cholinergic anti-inflammatory pathway via α7nAChR agonists [36] or bioelectronic vagal stimulation; and modulation of chemokine axes to disrupt leukocyte–nervous system crosstalk. Combining these approaches with established lipid-lowering agents and anti-inflammatory drugs holds promise for stabilizing vulnerable plaques and reducing adverse cardiovascular events [38, 112].

### Neuronal mediators in atherosclerosis and carotid stenosis

Neuronal markers such as tyrosine 3-monooxygenase or tyrosine hydroxylase (TH) and NE are essential for sympathetic signaling and pro-inflammatory responses within atherosclerotic plaques.

TH catalyzes the conversion of its substrates tyrosine and molecular oxygen to 3,4-dihydroxy-l-phenylalanine and represents the rate-limiting enzyme in the biosynthesis of catecholamines; among them, NE, also named noradrenaline, works as a hormone, neurotransmitter, and neuromodulator in the noradrenergic system.

In the vasculature, NE released from sympathetic nerve endings in the context of chronic inflammation binds to β-ARs on immune cells and VSMCs, triggering pathways that promote the production of inflammatory cytokines such as TNF-α and IL-1β [71, 72, 77]. In a rodent model, NE has also been demonstrated to induce VSMCs hyperproliferation and hypertension via adventitial fibroblast activation [109].

NE also increases MMP activity, which degrades the plaque’s fibrous cap, making it more susceptible to rupture and heightening the risk of ischemic events, particularly in carotid stenosis.

Both TH and NE have been implicated in vascular pathologies, including aneurysm formation and potentially atherosclerosis. Increased expression of TH in vascular tissues has been associated with SNS overactivation, leading to heightened oxidative stress, vascular inflammation, and ECM degradation. These processes contribute to endothelial dysfunction, VSMC remodeling, and chronic inflammation, key hallmarks of atherosclerosis. Pharmacological inhibition of TH has been shown to attenuate vascular remodeling by reducing MMP activity, preserving elastin integrity, and mitigating inflammatory responses. These findings suggest that targeting TH could represent a novel therapeutic strategy for vascular diseases, including atherosclerosis and aneurysm progression [11].

The NPY is a co-transmitter of the SNS widely expressed in the CNS and PeriphNS; at a physiological level, it has functions in the regulation of vascular tone and metabolism [52]. Its direct involvement in the pathogenesis of atherosclerosis has been studied since it promotes endothelial dysfunction, VSMCs proliferation, and local inflammatory responses, contributing to plaque growth and vulnerability [119]. In preclinical models, focal perivascular overexpression of NPY in ApoE-/- mice subjected to carotid collar results in a significant increase in plaque area and is associated with marked activation of perivascular mast cells. In carotid angioplasty rats and in mouse carotid/femoral injury models, NPY accelerates restenosis, while Y1 receptor blockade attenuates this response [52]. In humans, NPY expression is increased in unstable carotid plaques, and genetic variants of the NPY gene as well as elevated plasma levels have been associated with atherosclerosis, coronary artery disease, and ischemic stroke [52, 90, 119], suggesting it as a marker of atherosclerosis progression and vulnerability.

On the other hand, the PNS sustains the cholinergic anti-inflammatory pathway, a key mechanism for mitigating inflammatory responses: Ach released from the vagus nerve binds to α7nAChR on immune cells, blocking the production of inflammatory cytokines and promoting plaque stability; in line with this, the ANS deregulation in ischemic heart disease, along with neurostimulation techniques to restore the PNS/SNS equilibrium has been recently discussed [58]. A rodent model of atherosclerosis and AAA demonstrated the protective effect of α7nAChR activation by the selective agonist AR-R17779 [105], and this anti-inflammatory effect has prompted interest in VNS as a potential strategy to reduce chronic inflammation within atherosclerotic plaques [98]. By activating α7nAChR, VNS suppresses TNF-α, IL-1β, and other inflammatory mediators while enhancing Treg cells’ activity. Tregs, known for their role in maintaining immune homeostasis, secrete IL-10 and TGF-β, which are anti-inflammatory cytokines that contribute to plaque stabilization by inhibiting the activity of MMPs. This reduction in MMP activity preserves the integrity of the fibrous cap, lowering the risk of rupture and subsequent thromboembolic complications.

In the adventitia, the expression of GAP-43, an active neurite growth marker, has been observed in cells of the peripheral vascular system with a neuronal phenotype. These cells, in turn, contribute to the formation of a perivascular sensory neural network involved in the regulation of the arterial myogenic tone [94]. Recently, GAP-43 has been taken into consideration as a possible biomarker of disease severity in neurodegenerative conditions such as Alzheimer's disease [22, 88], leading to hypothesize its indirect connection in the context of neurovascular remodeling and peripheral neurovascular interactions.

Within NICIs, activation of TRPV1⁺ afferent sensory fibers leads to the release of the neuropeptide CGRP. CGRP has been shown to be a potent vasodilatory neuropeptide that suppresses the production of pro-inflammatory cytokines, notably TNF-α in macrophages and IL-12 in DC [91].

To date, the most compelling evidence for a protective role of CGRP against atherosclerosis has been obtained from murine models. In the context of atherosclerosis, CGRP promotes vasodilation by enhancing eNOS activity and inhibits NF-κB-dependent chemokine expression, such as CCL2 and CXCL8, thereby limiting monocyte adhesion and transmigration [103].

In a wire-induced right femoral artery procedure performed in wild-type and CGRP knockout (CGRP⁻/⁻) mice, these latter exhibited significantly increased vascular damage, characterized by enhanced neointimal formation, oxidative stress, macrophage infiltration, and VSMC migration into the neointima [108]. At the cellular level, in vitro, CGRP treatment restrained these effects [108]. In addition, in ApoE-/- mice, both CGRP-deficient mice and mice treated with the selective CGRP-neutralizing monoclonal antibody galcanezumab showed significantly increased atherosclerotic damage and lipid deposition at the aortic root [35]. Together, these findings support CGRP as a novel and promising candidate for cardiovascular protection.

SP has been identified as a neurogenic mediator between adventitia, mainly nerve fibers, and mast cell activation; in turn, mast cells are thought to be responsible for the release of pro-inflammatory, pro-hemorrhagic mediators causing plaque vulnerability and cardiovascular remodeling [54, 68]. Preclinical studies assessed the resistance to plaque vulnerability in mast cell-deficient ApoE-/- mice, establishing the critical involvement of mast cells in SP-elicited plaque destabilization [9], and indicated SP as a candidate neuroimmune target to restrain atherosclerosis complications (Table 1).

**Table 1:** Neuronal mediators/biomarkers in atherosclerosis

Neuronal mediators	Atherosclerotic disease	Preclinical model	Clinical setting	Ref
Tyrosine Hydroxylase (TH)	Promotes sympathetic activation, oxidative stress, inflammation, MMP activity; contributes to VSMC remodeling and plaque instability	ApoE-/- mice; TH inhibition reduces MMPs and vascular remodeling	TH is augmented in human vascular tissue associated with aneurysm and atherosclerosis	[11]
Norepinephrine (NE)	Triggers β-AR-mediated cytokine release (TNF-α, IL-1β), increases MMPs, promotes VSMC proliferation and plaque rupture risk	In ApoE-/- mice, NE activates adventitial fibroblasts and VSMCs	Promotes inflammation and plaque instability in carotid stenosis	[71, 72, 77, 109]
Neuropeptide Y (NPY)	Promotes endothelial dysfunction, VSMC proliferation, inflammation and foam cells formation; enhances plaque growth and vulnerability.	NPY promotes plaque growth, restenosis and mast cell activation in: ApoE-/- mice (carotid collar) with focal NPY overexpression. Rat carotid angioplasty and mouse carotid/femoral injury models. Metabolic stress (HFD/cold stress) models	NPY expression is higher in unstable human carotid plaque vs stable lesions; NPY polymorphisms are associated with atherosclerosis and cardiovascular risk	[52, 90, 119]
Acetylcholine (ACh)	Activates α7nAChR, anti-inflammatory mechanism; reduces cytokines, increases Tregs, stabilizes plaque	VNS or α7-nACh/ α7nAChR agonist (AR-R17779) in atherosclerotic mice, reduced inflammation and aneurysm	Explored in autonomic dysfunction and ischemic heart disease	[36, 58, 98]
α7 nicotinic ACh receptor (α7nAChR)	Target of ACh; mediates anti-inflammatory pathway (increases TNF-α, IL-1β, decreases IL-10, TGF-β, Tregs); stabilizes fibrous cap	ApoE-/- mice treated with AR-R17779; VNS shows plaque-protective effects	Candidate for neuromodulatory therapy (bioelectronic VNS)	[98, 105]
Growth Associated Protein-43 (GAP-43)	Marker of neural plasticity; suggests sensory nerve remodeling in inflamed lesions near ATLO	ApoE-/- mice with ATLO; GAP‑43⁺ NFM⁺ axon endings increased in adventitia adjacent to plaques; GAP-43 expressed in perivascular sensory cells (ANNIES) in rat mesenteric arteries	Not yet reported in clinical human plaque studies; potential imaging biomarker	[70, 94]
Calcitonin Gene-Related Peptide (CGRP)	Induces vasidilation; restrain VSMCs proliferation; limits inflammation and monocyte recruitment in atherosclerotic lesion	In mouse model of artery disease (wire injury; ApoE-/-), CGRP deficiency increases the damage entity	Serum CGRP levels negatively associate with coronary stenosis severity in patients without acute myocardial injury	[19, 35, 104, 108]
Substance P (SP)	Pro-inflammatory neuropeptide; promotes mast cell activation, cytokine release, angiogenesis, plaque vulnerability; dual role in acute vs chronic cardiovascular injury	ApoE-/- mice: SP induces adventitial mast cell activation and intraplaque emorrhage; cardioprotective in acute ischemia; promotes adverse remodeling via TNF-α/MMP release	Detected in human vulnerable plaques; elevated in heart failure patients; increased nerve-mast cell contacts in coronary atherosclerosis	[9, 54, 68]

### Role of ATLOs in neuroimmune modulation: present state of the art and therapeutic implications

ATLOs form in the adventitia of arteries in response to chronic inflammation and represent key sites of neuroimmune modulation. Within ATLOs, T and B cells, DCs, and macrophages actively interact, contributing either to plaque progression or stability. Th1 and Th17 cells within ATLOs produce IFN-γ and IL-17, respectively, which further drive inflammatory responses and increase plaque vulnerability. Conversely, Tregs and anti-inflammatory cytokines, such as IL-10 and TGF-β, stabilize the plaque by inhibiting MMP activity and promoting collagen deposition [74]. These ATLOs are directly influenced by sympathetic and parasympathetic signaling. Understanding the neuroimmune dynamics within ATLOs is essential for identifying new therapeutic targets to tackle plaque vulnerability by modulating pro- and anti-inflammatory signals.

Recent preclinical studies have also evaluated carotid body (CB) denervation as a means to ameliorate hypertension and its metabolic sequelae. In rodent models, resection of the carotid sinus nerve (CSN) not only reversed dysmetabolism and hypertensive phenotypes but also restored endothelial function in the aorta. Notably, CSN resection reduced NO levels in plasma and aortic tissue while normalizing inducible NO synthase expression in the aorta, without affecting eNOS or prostaglandin F2α receptor levels. These findings indicate that modulation of CB activity may offer a novel avenue for treating hypertension and endothelial dysfunction, particularly in the context of type 2 diabetes mellitus [10].

There is growing interest in therapeutic approaches targeting the ANS to mitigate vascular damage. The rationale stems from increasing evidence linking neuroimmune interactions with vascular inflammation and remodeling: given the complex interactions within the CBI axis, several therapies aim to modulate specific neuroimmune pathways to achieve anti-inflammatory effects and hamper atherosclerotic progression. For instance, the use of β-adrenergic blockers, which inhibit sympathetic activity by blocking AR and reducing NE levels. β-Adrenergic blockers, already employed to lower blood pressure and cardiovascular risk, appear to also limit pro-inflammatory cytokine production and MMP activity within plaques [37, 65, 83]. VNS, with the potential to activate the cholinergic anti-inflammatory pathway, reduces pro-inflammatory cytokines and enhances Treg function within plaques. VNS stabilization is linked to a decrease in MMP production and an increase in IL-10 and TGF-β levels, effectively lowering plaque rupture risk [3, 96]. Carotid baroreflex modulation via implantable devices represents a promising therapeutic option for drug-resistant hypertension and heart failure [41, 63]. Renal denervation, originally developed to treat resistant hypertension, has shown potential in reducing systemic sympathetic activity, which is known to exacerbate endothelial dysfunction and promote vascular inflammation [6, 93]. Similarly, cardiac denervation techniques are being explored to attenuate autonomic imbalance and reduce the risk of arrhythmias and heart failure progression, both of which contribute to adverse vascular outcomes [84]. These neuro-modulatory strategies may intersect with the formation and function of ATLOs.

Although still largely theoretical, several studies have suggested that atherosclerosis could be targeted through TRPV agonists. In vivo, long-term activation of TRPV1 by capsaicin in the aorta of ApoE-/- mice attenuates high-fat diet-induced atherosclerosis [62]. Similarly, the TRPV1 agonist evodiamine has been shown to counteract atherosclerosis progression by alleviating endothelial cell inflammation [103].

By influencing sympathetic and parasympathetic tone or sensory fiber activation, such interventions could potentially affect the local immune environment, altering cytokine profiles and immune cell trafficking in ways that modulate ATLO activity. While still largely unexplored, the link between autonomic regulation and vascular immune responses opens new avenues for integrated therapies aimed at both the nervous and immune systems to prevent or limit atherosclerotic progression [29].

### Future directions in neuroimmune-based therapy

The CBI axis represents a rapidly expanding field in atherosclerosis research. Understanding the neuroimmune details within plaques and the dynamics of ATLO and nervous system interactions could lead to increasingly targeted therapeutic approaches. Advanced imaging technologies, such as PET with tracers specific for NE and Ach, allow for real-time visualization of neuronal and immune activity, providing a comprehensive view of site-specific plaque instability risk. This new perspective of analysis may greatly improve the diagnosis of disease stage and rate of progression, increasing in this way the criteria of analysis and improving the risk stratification of patients.

Finally, personalized neuroimmune modulation may open new avenues for treating carotid stenosis. Targeted therapies that balance sympathetic and parasympathetic signals, or reinforce immune regulation within ATLOs, could lead to a significant reduction in plaque rupture risk and stroke incidence in patients with advanced atherosclerosis. As research progresses, an integrated approach that includes neuroimmune modulators and traditional lipid-lowering therapies could greatly improve the clinical management of carotid stenosis.

## Concluding remarks

In conclusion, ATLOs represent pivotal sites of neuroimmune interaction within atherosclerotic plaques, especially in carotid stenosis where plaque instability can have devastating consequences. By integrating local immune regulation with neural signaling via sympathetic and parasympathetic systems, ATLOs influence the balance between inflammation and resolution. Their dual role, as drivers of both pro- and anti-inflammatory responses, positions them as key determinants of plaque stability. Emerging therapies that modulate neuroimmune pathways, such as VNS or beta-blockers, show promise in shifting ATLO function toward stabilization. Advanced imaging modalities further support the potential for non-invasive assessment of ATLO activity and neuroimmune dynamics. As understanding deepens, ATLOs are increasingly viewed not just as bystanders or biomarkers, but as active therapeutic targets. Their modulation could complement existing approaches to reduce atherosclerosis-related major events, such as stroke risk and improve clinical outcomes in patients with progressive atherosclerosis.

## Abbreviations

AAA: Abdominal aortic aneurysm
Ach: Acetylcholine
ARs: Adrenergic receptors
AGEs: Advanced glycation end products
α7nAChR: Alpha 7 nicotinic acetylcholine receptors
ApoE-/-: Apolipoprotein E-deficient
ABC: Artery–brain circuit
ATLOs: Artery tertiary lymphoid organs
ANS: Autonomic nervous system
CGRP: Calcitonin gene-related peptide
CBI: Cardiovascular-brain-immune
CB: Carotid body
CSN: Carotid sinus nerve
CNS: Central nervous system
DCs: Dendritic cells
eNOS: Endothelial nitric oxide synthase
ECM: Extracellular matrix
FRCs: Fibroblastic reticular cells
FDC: Follicular dendritic cell
Tfr: Follicular regulatory T
GCs: Germinal centres
GAP-43: Growth-associated protein 43
HEVs: High endothelial venules
IgG4-AAA: IgG4-related abdominal aortic aneurysms
LTβR: Lymphotoxin-β receptor
MMPs: Matrix metalloproteinases
MI: Myocardial infarction
PeriphNS: Peripheral nervous system
NICIs: Neuroimmune cardiovascular interfaces
NK1R: Neurokinin 1 receptor
NPY: Neuropeptide Y
NO: Nitric oxide
NE: Norepinephrine
NFAT: Nuclear factor of activated T cells
oxLDL: Oxidized low-density lipoproteins
PNS: Parasympathetic nervous system
PTLOs: Plaque tertiary lymphoid organs
PET: Positron emission tomography
PLOs: Primary lymphoid organs
RAMP1/CLR: Receptor activity-modifying protein 1 and calcitonin receptor-like receptor
Tregs: Regulatory T cells
SLOs: Secondary lymphoid organs
SMCs: Smooth muscle cells
SNS: Sympathetic nervous system
SP: Substance P
Tfh: T follicular helper
TLOs: Tertiary lymphoid organs
TH: Tyrosine hydroxylase
TRP: Transient receptor potential
TRPV: Transient receptor potential vanilloid
TNFα: Tumor necrosis factor-alpha
VN: Vagus nerve
VNS: Vagus nerve stimulation
VSMCs: Vascular smooth muscle cells
